# Nomogram for predicting postoperative cancer-specific early death in patients with epithelial ovarian cancer based on the SEER database: a large cohort study

**DOI:** 10.1007/s00404-021-06342-x

**Published:** 2021-11-29

**Authors:** Tingting Zhang, Liancheng Zhu

**Affiliations:** Department of Obstetrics and Gynecology, Shengjing Hospital of China Medical University, Shenyang, 110004 Liaoning China

**Keywords:** Ovarian cancer, Nomogram, Prognosis, Cancer-specific survival, Early death, Surveillance, Epidemiology, End Results

## Abstract

**Purpose:**

Ovarian cancer is a common gynecological malignant tumor. Poor prognosis is strongly associated with early death, but there is no effective tool to predict this. This study aimed to construct a nomogram for predicting cancer-specific early death in patients with ovarian cancer.

**Methods:**

We used data from the Surveillance, Epidemiology, and End Results database of patients with ovarian cancer registered from 1988 to 2016. Important independent prognostic factors were determined by univariate and multivariate logistic regression and LASSO Cox regression. Several risk factors were considered in constructing the nomogram. Nomogram discrimination and calibration were evaluated using C-index, internal validation, and receiver operating characteristic (ROC) curves.

**Results:**

A total of 4769 patients were included. Patients were assigned to the training set (*n* = 3340; 70%) and validation set (*n* = 1429; 30%). Based on the training set, eight variables were shown to be significant factors for early death and were incorporated in the nomogram: American Joint Committee on Cancer (AJCC) stage, residual lesion size, chemotherapy, serum CA125 level, tumor size, number of lymph nodes examined, surgery of primary site, and age. The concordance indices and ROC curves showed that the nomogram had better predictive ability than the AJCC staging system and good clinical practicability. Internal validation based on validation set showed good consistency between predicted and observed values for early death.

**Conclusion:**

Compared with predictions made based on AJCC stage or residual lesion size, the nomogram could provide more robust predictions for early death in patients with ovarian cancer.

## Introduction

Ovarian cancer is the most common malignant tumor of the female reproductive system. Though relatively rare, with an incidence of 0.0119% [1], it is the seventh most common cancer in women [2] and has a high recurrence risk. It also has the second highest mortality rate and the worst prognosis among the major gynecologic cancers [3, 4] (endometrial cancer, cervical cancer, and ovarian cancer). Its 5-year survival rate after diagnosis varies widely among countries, at 46% in the United States [5] and 26–51% elsewhere [6]. According to US and UK studies, the mortality-to-morbidity ratio of ovarian cancer is greater than 0.6, with one in six women dying within 90 days after diagnosis [3]. Epithelial ovarian cancer (EOC), the most common type of ovarian cancer [7], accounts for more than 95% of ovarian malignant tumors. Compared with other types, it has higher incidence and mortality rates and has different histopathological features, including cell sources, morphology, molecular characteristics, epidemiological factors, clinical characteristics, and survival patterns. Sixty percent of EOC patients develop distant disease, and the average 5-year survival rate is only 29% [1].

Early-stage ovarian cancer has no symptoms, and no methods can effectively monitor them. The primary treatment is surgery and postoperative chemotherapy, which are considerable burdens to the patient. Identifying high-risk factors for ovarian cancer can provide individualized advice and guidance for surgical procedures early in the diagnosis. Ovarian cancer also has a high early death (ED) rate, and exploring factors related to ED can help clinicians identify high-risk patients and develop targeted treatment to improve their survival and quality of life. Therefore, it is necessary to establish an ED prediction model for ovarian cancer to help gynecological oncologists individualize treatments. To our knowledge, no study has been conducted for predicting ED in ovarian cancer postoperatively.

Nomograms are widely used tools that predict incidence and prognosis by combining multiple variables in a single chart. Gynecologists have consistently aimed to improve ovarian cancer survival rates and have recently shown interest in nomograms. However, there are currently no nomograms for the visual prediction, especially postoperatively, of ED in EOC. Although there are studies on the prognosis of malignant tumors, most have focused on long-term survival. Few studies focused on ED, and these were based on small samples or regionally limited cohorts [6, 8, 9].

The Surveillance, Epidemiology, and End Results (SEER) database is an authoritative source of information on cancer incidence and survival status in the United States (https://seer.cancer.gov), representing a sum of statistics from population-based registries that cover over one-third of the US population. Unlike single-center studies, the SEER registry publishes and regularly updates extensive data on patient demographics, primary tumor site, tumor morphology, disease extent, first course of treatment, and active follow-up for vital status. In this study, we used the SEER database to evaluate related factors and construct a nomogram for predicting ED in EOC.

## Materials and methods

### Ethics statement

This study used the SEER database and does not require informed consent. Standard ethical standards were met.

### Study population

**Fig. 1:**
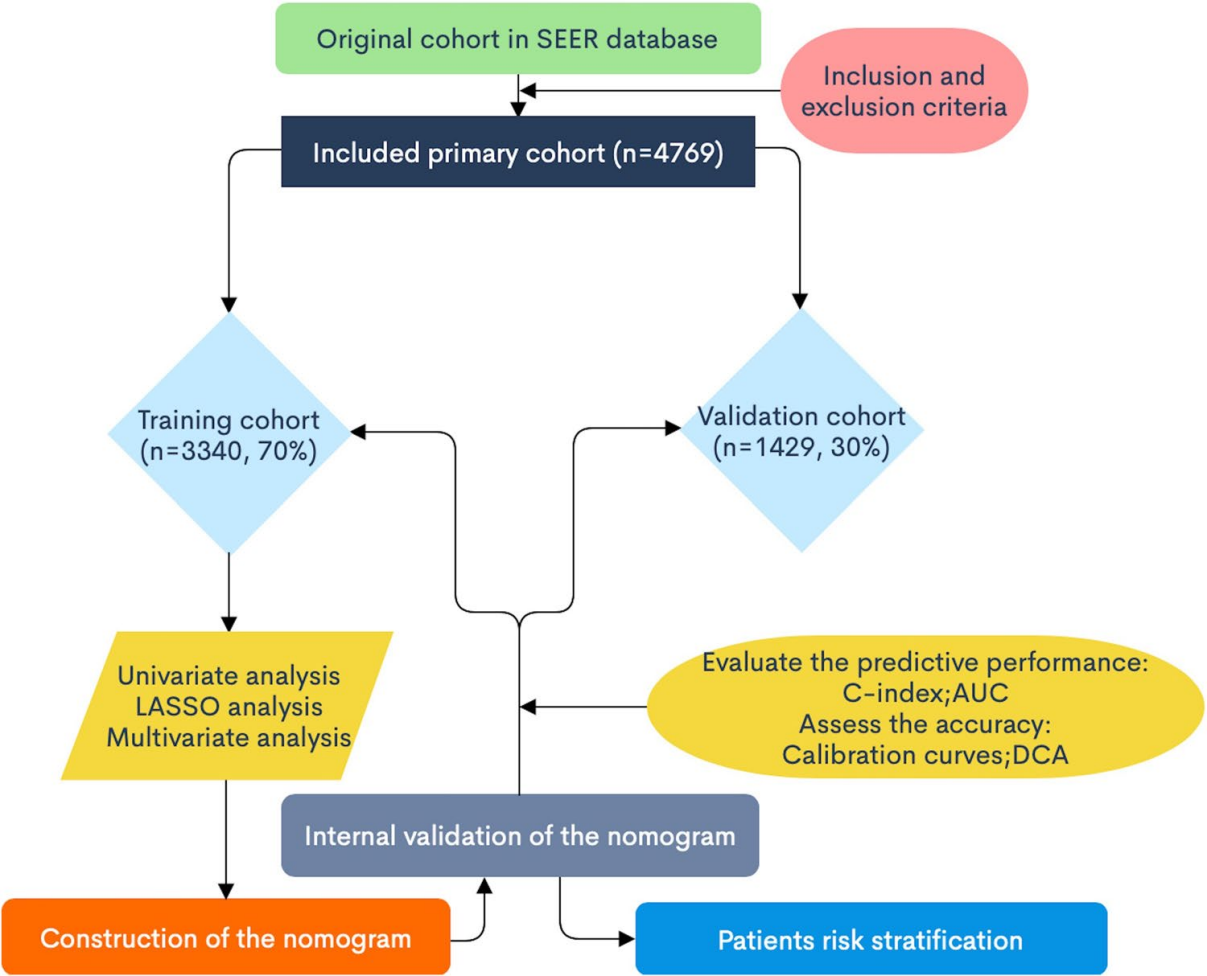
Patient selection flowchart. *SEER* Surveillance, Epidemiology, and End Results

We used SEER *Stat version 8.3.9 to extract data on EOC patients between 1988 and 2016. The inclusion criteria were the site code for ovary (C56.9) and histology codes: Serous (8441–8442, 8460–8463, 9014); Mucinous (8144, 8384, 8470–8472, 8480–8482); Endometrioid (8380–8383); Clear cell (8310, 8313, 8443–8444, 9110); Transitional cell (8120, 8122, 8130, 9000); and Epithelial-stromal (8800–8801, 8804–8805, 8810, 8814, 8840, 8850–8851, 8854, 8890–8891, 8896, 8900–8902, 8920–8921, 8930–8931, 8933, 8935, 8936, 8950), according to the International Classification of Tumor Diseases, Third Edition (ICD-O-3). The exclusion criteria were (1) unknown cause of death, (2) unknown survival time, (3) unknown tumor size, (4) unknown lymph node information, (5) unknown surgical treatment, (6) unknown race, and (7) unknown staging. Figure 1 shows the patient selection criteria flowchart.

“Early-stage” ovarian cancer death is not clearly defined in the literature. Urban et al. [8] defined it as 3 months based on economic definitions, whereas Mosgaard et al. [6] and Lefur et al. [9] defined it as 6 months. Therefore, in our study, we included postoperative patients with a follow-up of ≤ 6 months and studied ED from ovarian cancer at 1, 3, and 6 months.

### Data collection

Patient demographics and clinical characteristics were extracted from the SEER database, including age, race, marital status, AJCC stage, laterality, surgery of primary site, chemo- and radiotherapy, regional lymph node status (number examined and positive or negative), tumor size, serum CA125 level, residual lesion size, grade, histological type, and metastases to the bone, brain, liver, and lung. We focused on cancer-specific ED.

### Statistical analysis and nomogram construction

**Fig. 2:**
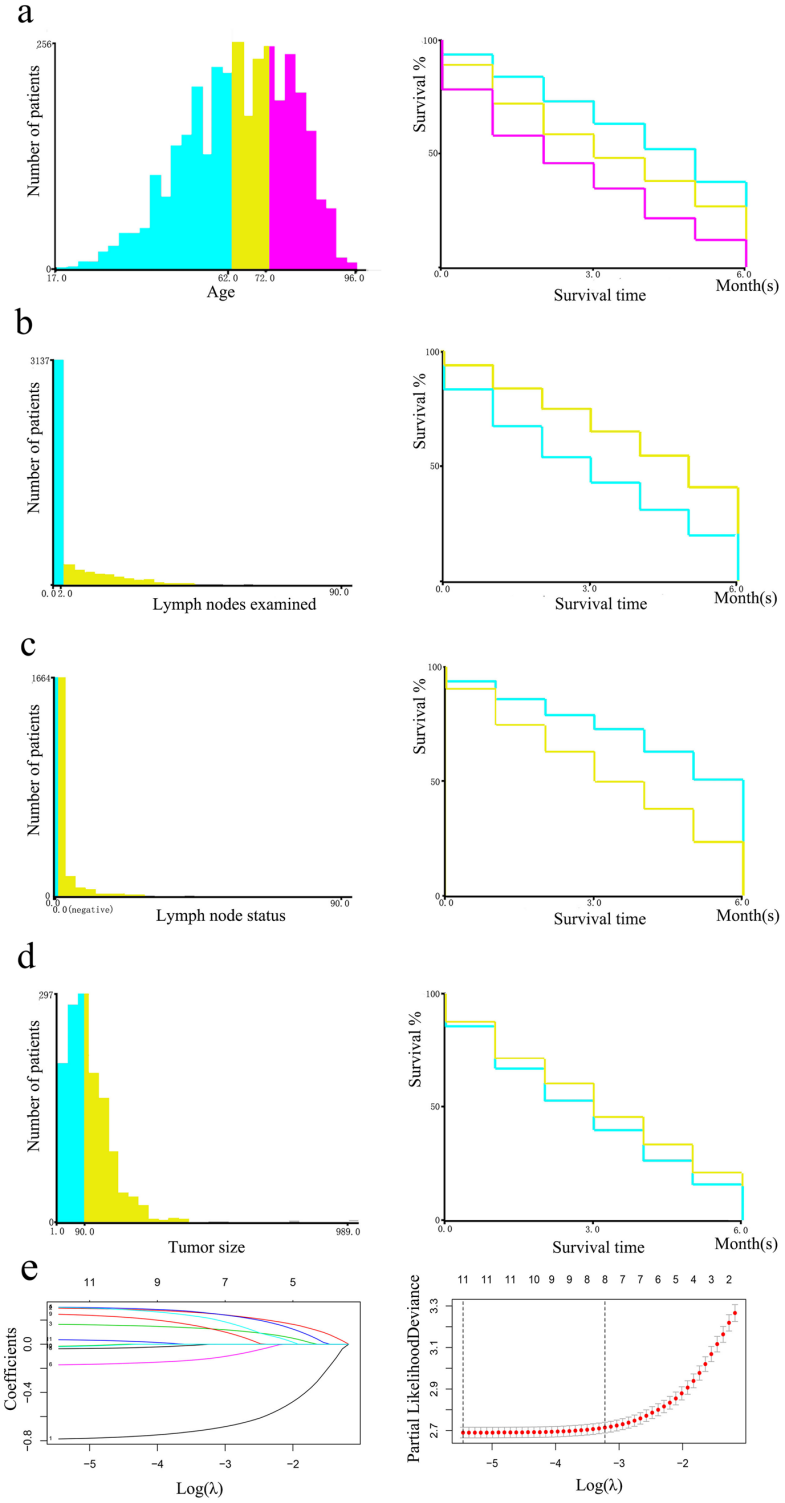
Cutoff values for age, lymph node examined, lymph node status, and tumor size that were assessed by X-tile analysis. (**a**) The appropriate cutoff values for age were 62 and 72 years old (*P* < 0.001). (**b**) The appropriate cutoff value for the number of lymph nodes examined was 2 (*P* < 0.001). (**c**) The appropriate cutoff value of lymph node status was positive or negative (*P* < 0.001). (**d**) The appropriate cutoff value for tumor size was 90 mm (*P* < 0.001). (**e**) LASSO regression identified 12 variables for CSS and LASSO Cox analysis identified 11 variables for CSS. CSS: cancer-specific survival

X-tile was used to stratify age, number of lymph nodes examined, number of positive lymph nodes, and tumor size [10]. The cutoff values of age were 62 and 72 years; number of lymph nodes examined, 2; lymph nodes status, positive or negative; and tumor size, 90 mm (Fig. 2a–d). Patients included in the study were divided into the training set and validation set at a ratio of 7:3. R (version 3.6.0) was used to analyze all data in an R Studio environment. Univariate Cox regression was used to assess the factors associated with ED. Variables that showed statistical significance (*P* < 0.05) were included in the LASSO regression analysis.

Variables that remained significant after LASSO regression analysis were included in the multivariate Cox regression analysis. A *P* value of < 0.01 was used as the modeling index. The rms package in R software was used to establish the nomogram for predicting ED based on the associated risk factors in patients with ovarian cancer. The pROC package was used to generate ROC curves. The nomogram was internally validated in the validation set based on the area under curve (AUC). The original data and validation model were compared using the concordance index (C-index) to evaluate calibration accuracy. Nomogram discrimination was measured with the C-index at 95% confidence level, which quantifies the degree of agreement between the prediction probability and actual occurrence probability. A larger C-index indicates a more accurate prediction of prognosis. The primary terminus of the observed event was cancer-specific ED. The classified variables are presented as frequency (%), hazard ratio, and 95% confidence interval (CI).

## Results

### Patient characteristics and survival outcomes

Based on the inclusion and exclusion criteria, 4769 patients were included in the study. ED occurred in most patients who were white (83.58%). Most patients had no metastasis, and all patients underwent surgery. Fifty-nine patients (1.24%) underwent radiotherapy and 2610 (54.73%) underwent chemotherapy. The patients were divided into the training set (*n* = 3340; 70%) and the validation set (*n* = 1429; 30%). Patient characteristics for both sets are shown in Table 1.

**Table 1:** Characteristics with epithelial ovarian Patients

Characteristic	All patients, *n* (%)	Training set, *n* (%)	Validation set, *n* (%)	*P* value	SMD	Missing
Total	4769 (100.0)	3340 (70.0)	1429 (30.0)			
Age, years				0.555	0.034	0
17–62	2021 (42.4)	1426 (42.7)	595 (41.6)			
63–72	1160 (24.3)	818 (24.5)	342 (23.9)			
73–96	1588 (33.3)	1096 (32.8)	492 (34.4)			
Race				0.638	0.051	0
White	3986 (83.6)	2784 (83.4)	1202 (84.1)			
Black	347 (7.3)	254 (7.6)	93 (6.5)			
Asian	387 (8.1)	269 (8.1)	118 (8.3)			
American Indian	30 (0.6)	19 (0.6)	11 (0.8)			
Unknown	19 (0.4)	14 (0.4)	5 (0.3)			
Marital status				0.757	0.043	0
Single	839 (17.6)	596 (17.8)	243 (17.0)			
Married	2204 (46.2)	1544 (46.2)	660 (46.2)			
Widowed/Separated	1099 (23.1)	755 (22.6)	344 (24.1)			
Divorced	464 (9.7)	332 (9.9)	132 (9.2)			
Unknown	163 (3.4)	113 (3.4)	50 (3.5)			
AJCC stage				0.842	0.029	0
I	676 (14.2)	482 (14.4)	194 (13.6)			
II	313 (6.6)	215 (6.4)	98 (6.9)			
III	2134 (44.7)	1490 (44.6)	644 (45.1)			
IV	1646 (34.5)	1153 (34.5)	493 (34.5)			
Laterality				0.227	0.054	0
Unilateral	2479 (52.0)	1756 (52.6)	723 (50.6)			
Paired	197 (4.1)	129 (3.9)	68 (4.8)			
Bilateral	2093 (43.9)	1455 (43.6)	638 (44.6)			
Surgery of primary site				0.499	0.058	0
Fertility-sparing	905 (19.0)	624 (18.7)	281 (19.7)			
Non-fertility-sparing	438 (9.2)	317 (9.5)	121 (8.5)			
Debulking	1334 (28.0)	949 (28.4)	385 (26.9)			
Pelvic exenteration	2092 (43.8)	1450 (43.4)	642 (44.9)			
Chemotherapy				0.463	0.024	0
None/Unknown	2159 (45.3)	1500 (44.9)	659 (46.1)			
Yes	2610 (54.7)	1840 (55.1)	770 (53.9)			
Number of lymph nodes examined				0.273	0.05	0
No regional nodes removed/ number of nodes is unknown	166 (3.5)	107 (3.2)	59 (4.1)			
0–2	3006 (63.0)	2108 (63.1)	898 (62.8)			
≥ 3	1597 (33.5)	1125 (33.7)	472 (33.0)			
Tumor size				0.497	0.038	0
Unknown	3419 (71.7)	2379 (71.2)	1040 (72.8)			
≤ 90 mm	698 (14.6)	493 (14.8)	205 (14.3)			
> 90 mm	652 (13.7)	468 (14.0)	184 (12.9)			
Serum CA125 level				0.662	0.029	0
Negative	206 (4.3)	150 (4.5)	56 (3.9)			
Borderline	2337 (49.0)	1631 (48.8)	706 (49.4)			
Positive	2226 (46.7)	1559 (46.7)	667 (46.7)			
Residual lesion size				0.926	0.022	0
No residual lesion	1027 (21.5)	725 (21.7)	302 (21.1)			
≤ 1 cm	224 (4.7)	160 (4.8)	64 (4.5)			
> 1 cm	160 (3.4)	112 (3.4)	48 (3.4)			
Residual unknown	3358 (70.4)	2343 (70.1)	1015 (71.0)			
Lymph node status				0.11	0.075	0
No nodes examined/unknown status	2665 (55.9)	1860 (55.7)	805 (56.3)			
Unknown number of positive nodes	79 (1.7)	47 (1.4)	32 (2.2)			
Negative	1127 (23.6)	809 (24.2)	318 (22.3)			
Positive	898 (18.8)	624 (18.7)	274 (19.2)			
Grade				0.44	0.052	0
I	382 (8.0)	277 (8.3)	105 (7.3)			
II	824 (17.3)	580 (17.4)	244 (17.1)			
III	2352 (49.3)	1654 (49.5)	698 (48.8)			
IV	1211 (25.4)	829 (24.8)	382 (26.7)			
Histological type				0.412	0.071	0
Serous	2956 (62.0)	2041 (61.1)	915 (64.0)			
Mucinous	595 (12.5)	431 (12.9)	164 (11.5)			
Endometrioid	565 (11.8)	406 (12.2)	159 (11.1)			
Clear cell	314 (6.6)	217 (6.5)	97 (6.8)			
Transitional	22 (0.5)	15 (0.4)	7 (0.5)			
Epithelial-stromal	317 (6.6)	230 (6.9)	87 (6.1)			
Radiotherapy				0.736	0.015	0
None/Unknown	4710 (98.8)	3297 (98.7)	1413 (98.9)			
Yes	59 (1.2)	43 (1.3)	16 (1.1)			
Bone metastasis				0.956	0.01	0
None/Unknown	4751 (99.6)	3328 (99.6)	1423 (99.6)			
Yes	18 (0.4)	12 (0.4)	6 (0.4)			
Brain metastasis				0.449	0.038	0
None/Unknown	4766 (100.0)	3339 (100.0)	1427 (99.9)			
Yes	3 (0.00)	1 (0.0)	2 (0.1)			
Liver metastasis				0.421	0.029	0
None/Unknown	4619 (96.9)	3230 (96.7)	1389 (97.2)			
Yes	150 (3.1)	110 (3.3)	40 (2.8)			
Lung metastasis				0.705	0.016	0
None/Unknown	4675 (98.0)	3272 (98.0)	1403 (98.2)			
Yes	94 (2.0)	68 (2.0)	26 (1.8)			

*SMD* standardized mean difference; *AJCC* American Joint Committee on Cancer

### Risk factor analysis for ED

Table 2 shows the results of the univariate Cox regression analysis. Twelve characteristics showed a higher risk of cancer-specific ED, including older age, black, married, stage IV, bilateral disease, fertility-sparing surgery, no chemotherapy, no lymph nodes removed, larger tumor size, positive serum CA125 level, larger residual lesion size, and positive lymph nodes (*P* < 0.05). LASSO Cox regression analysis with the above variables showed that the following were involved in the composition of cancer-specific survival (CSS) risk factors: age, race, marital status, AJCC stage, laterality, surgery of primary site, chemotherapy, number of lymph nodes examined, tumor size, serum CA125 level, and residual lesion size (Fig. 2e). Multivariate Cox regression analysis showed a higher rate of cancer-specific ED for patients who were older, black, and married and had undergone no or unknown chemotherapy, large residual lesion size, higher stage disease, positive CA125 level, no or unknown number of lymph nodes examined, undergone fertility-sparing surgery, larger tumor size, and bilateral onset (Table 2).

**Table 2:** Univariate and multivariate Cox regression analysis of cancer-specific mortality

Characteristic	Univariate cox regression			Multivariate cox regression		
	HR	95% CI	*P* value	HR	95% CI	*P* value
Age, years	1.599	1.522–1.679	< 0.001			
17–62				Reference		
63–72				1.175	1.047–1.318	0.006096
73–96				1.342	1.194–1.509	< 0.001
Race	0.859	0.803–0.919	0.002			
White				Reference		
Black				1.080	0.922–1.264	0.340927
Asian				0.959	0.804–1.144	0.640988
American Indian				0.799	0.470–1.360	0.409096
Unknown				0.288	0.072–1.155	0.05
Marital status	1.115	1.072–1.161	< 0.001			
Single				Reference		
Married				1.014	0.891–1.154	0.827977
Widowed/Separated				0.993	0.857–1.151	0.925082
Divorced				0.916	0.769–1.090	0.321994
Unknown				0.760	0.581–0.996	0.046391
AJCC stage	1.568	1.492–1.648	< 0.001			
I				Reference		
II				2.233	1.677–2.972	< 0.001
III				3.220	2.571–4.033	< 0.001
IV				3.596	2.862–4.519	< 0.001
Laterality	1.101	1.055–1.148	< 0.001			
Unilateral				Reference		
Paired				0.932	0.757–1.147	0.504569
Bilateral				1.054	0.964–1.153	0.248700
Surgery of primary site	0.828	0.799–0.859	< 0.001			
Fertility-sparing				Reference		
Non-fertility-sparing				0.840	0.709–0.995	0.043971
Debulking				0.651	0.569–0.744	< 0.001
Pelvic exenteration				0.750	0.667–0.844	< 0.001
Chemotherapy	0.352	0.324–0.383	< 0.001			
None/Unknown				Reference		
Yes				0.391	0.357–0.430	< 0.001
Number of lymph nodes examined	0.537	0.495–0.582	< 0.001			
No regional nodes removed/ number of nodes is unknown				Reference		
0–2				0.783	0.639–0.958	0.017434
≥ 3				0.637	0.512–0.792	< 0.001
Tumor size	1.185	1.124–1.249	< 0.001			
Unknown				Reference		
≤ 90 mm				1.700	1.499–1.927	< 0.001
> 90 mm				1.773	1.561–2.013	< 0.001
Serum CA125 level	1.991	1.841–2.154	< 0.001			
Negative				Reference		
Borderline				1.290	0.932–1.787	0.125217
Positive				1.858	1.339–2.580	< 0.001
Residual lesion size	1.715	1.632–1.801	< 0.001			
No residual lesion				Reference		
≤ 1 cm				1.355	1.019–1.800	0.036478
> 1 cm				1.901	1.388–2.605	< 0.001
Residual unknown				2.718	2.296–3.218	< 0.001
Lymph node status	0.817	0.788–0.846	< 0.001			
Grade	1.001	0.956–1.049	0.959			
Histological type	1.003	0.975–1.032	0.845			
Radiotherapy	0.969	0.684–1.374	0.860			
Bone metastasis	0.825	0.412–1.652	0.587			
Brain metastasis	2.456	0.346–17.453	0.369			
Liver metastasis	0.856	0.665–1.102	0.229			
Lung metastasis	0.808	0.589–1.109	0.188			

*HR* hazard ratio; *CI* confidence interval; *AJCC* American Joint Committee on Cancer

### Nomogram construction

**Fig. 3:**
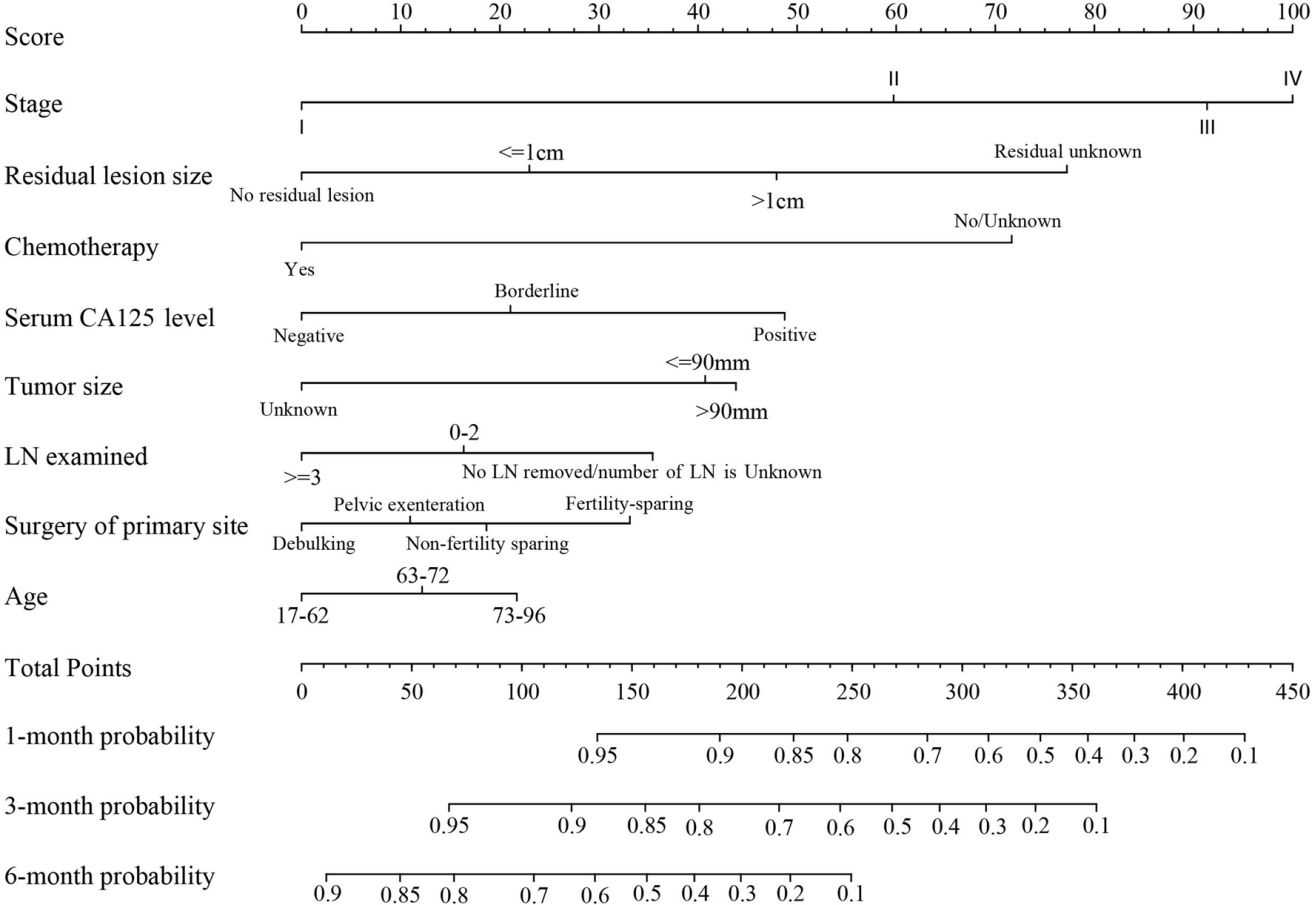
Nomogram for predicting 1-, 3-, and 6-month CSS. LN: lymph node; CSS: cancer-specific survival

Multivariate regression analysis revealed that AJCC stage, residual lesion size, chemotherapy, serum CA125 level, tumor size, number of lymph nodes examined, surgery of primary site, and age were significantly associated to ED (*P* < 0.05). These eight variables were used to construct a nomogram for predicting cancer-specific ED (Fig. 3). The procedure for using the nomogram is as follows: based on the patient’s status, a vertical line is drawn from each prediction variable to the “Score” axis. Each prediction variable is then assigned the corresponding points shown by the intersection of the vertical line with the “Score” axis. The points from all variables are added to obtain the total points, which is used to draw another vertical line from the “Total Points” axis to the probability axes. The intersection of this line with the 1-, 3-, and 6-month axes shows the probability of cancer-specific ED for those time intervals. The C-indices of the training and validation set were 0.787 (95%CI: 0.772–0.794), 0.763 (95%CI: 0.745–0.780), respectively, indicating good consistency between the predicted and observed values.

### Performance of nomogram

**Fig. 4:**
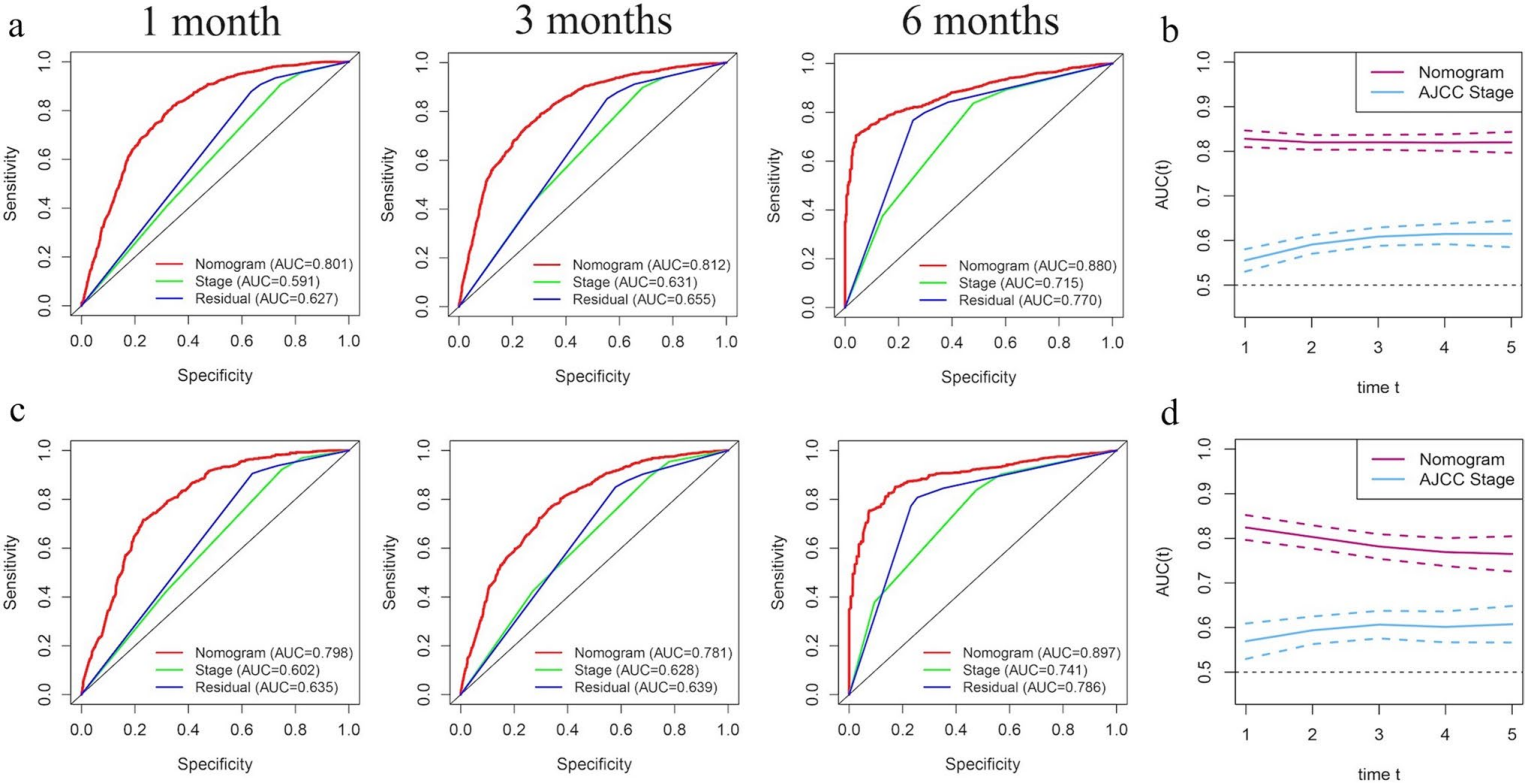
ROC curves for the nomogram, stage, and residual lesion size predictions of cancer-specific early death in the (**a**) training set and (**c**) validation set for 1-, 3-, and 6-month survival. Time dependence of the AUC for the nomogram and AJCC stage predictions of early death in the (**b**) training set and (**d**) validation set. *AUC* area under the curve; *ROC* receiver operating characteristic

**Fig. 5:**
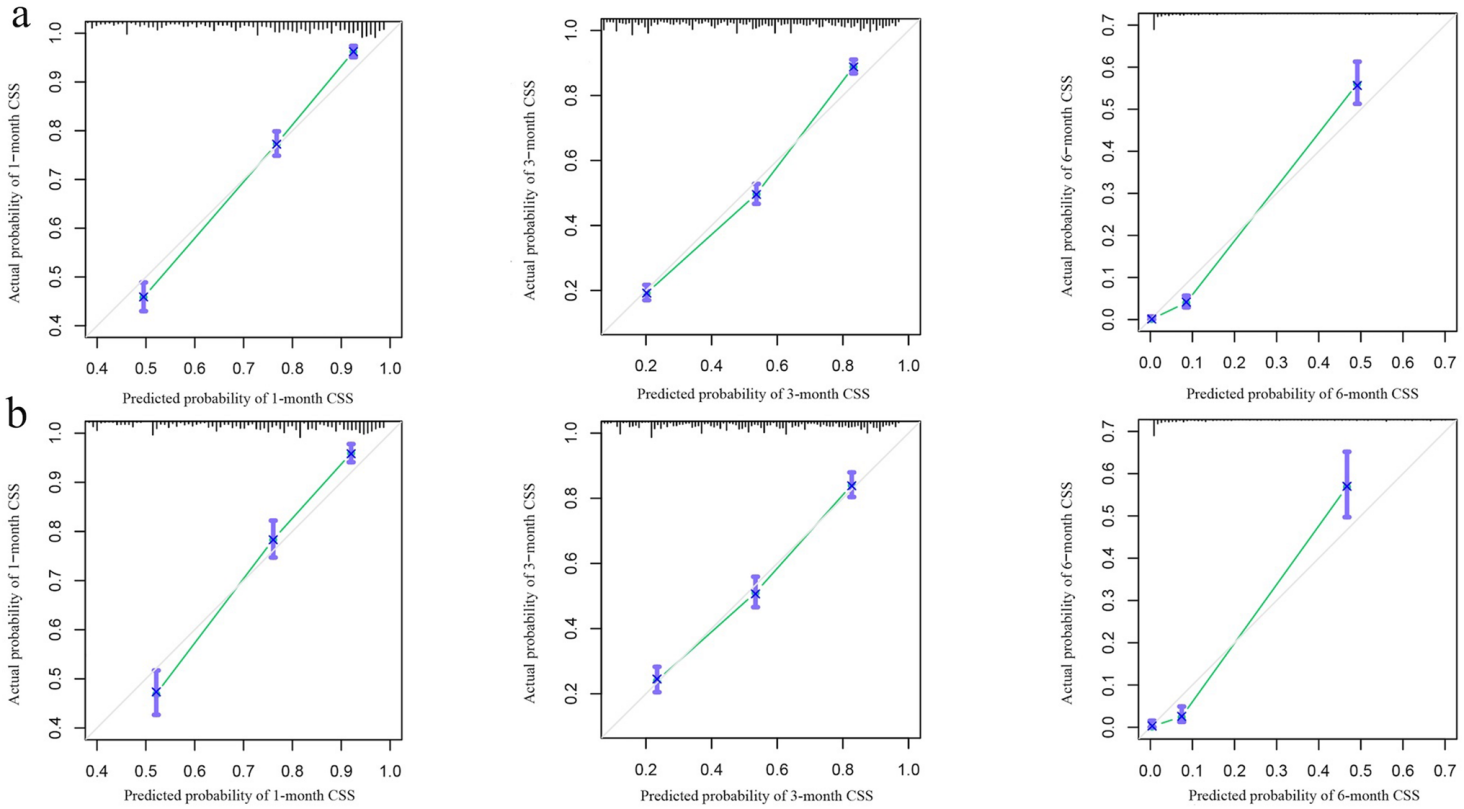
Internal validation plots for evaluating nomogram calibration, at 1-, 3-, and 6-month cancer-specific survival (CSS). (**a**) Training set, 1, 3, and 6 months. (**b**) Validation set, 1, 3, and 6 months

**Fig. 6:**
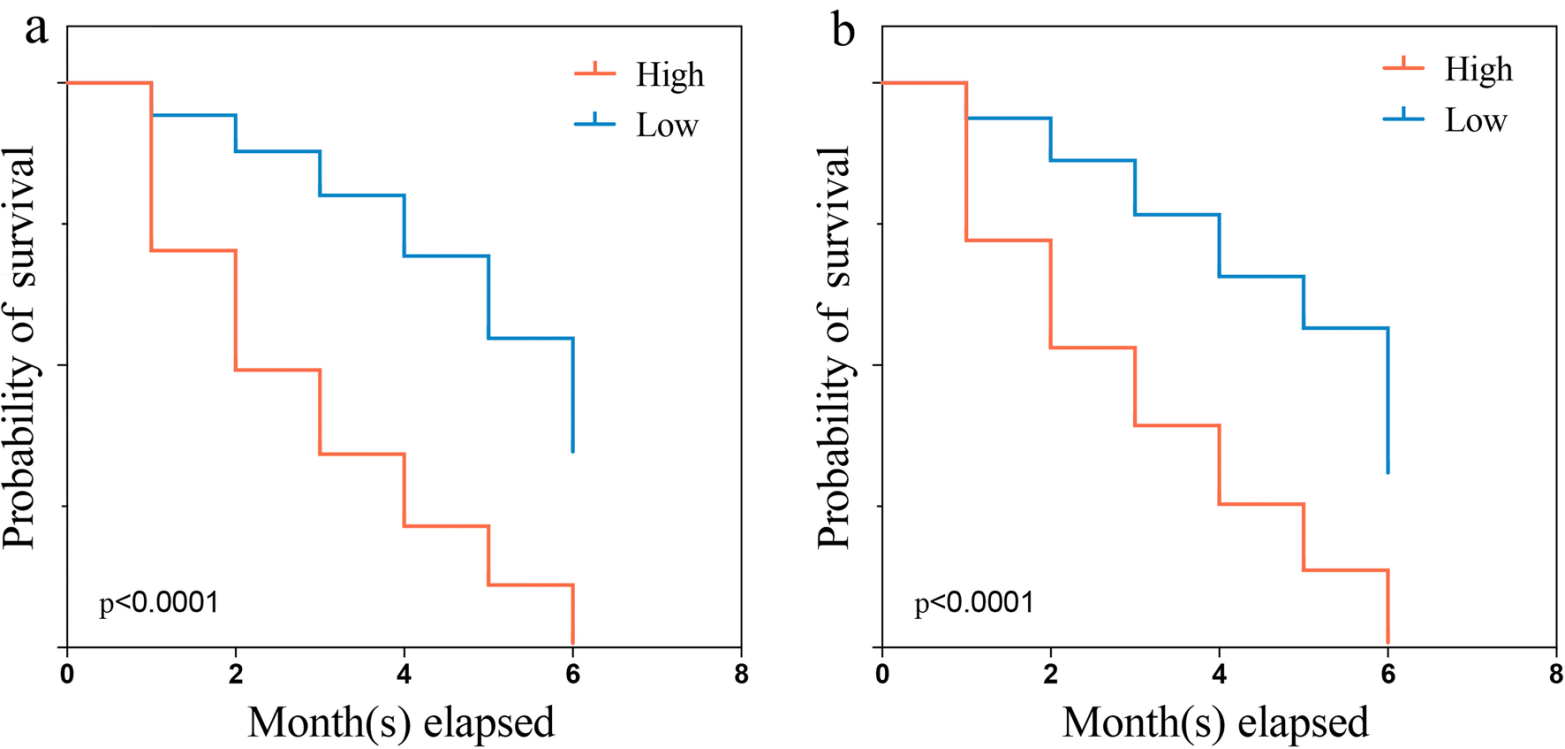
Kaplan–Meier curves showing cancer-specific survival of patients stratified by the risk stratification system. (**a**) Training set. (**b**) Validation set

**Fig. 7:**
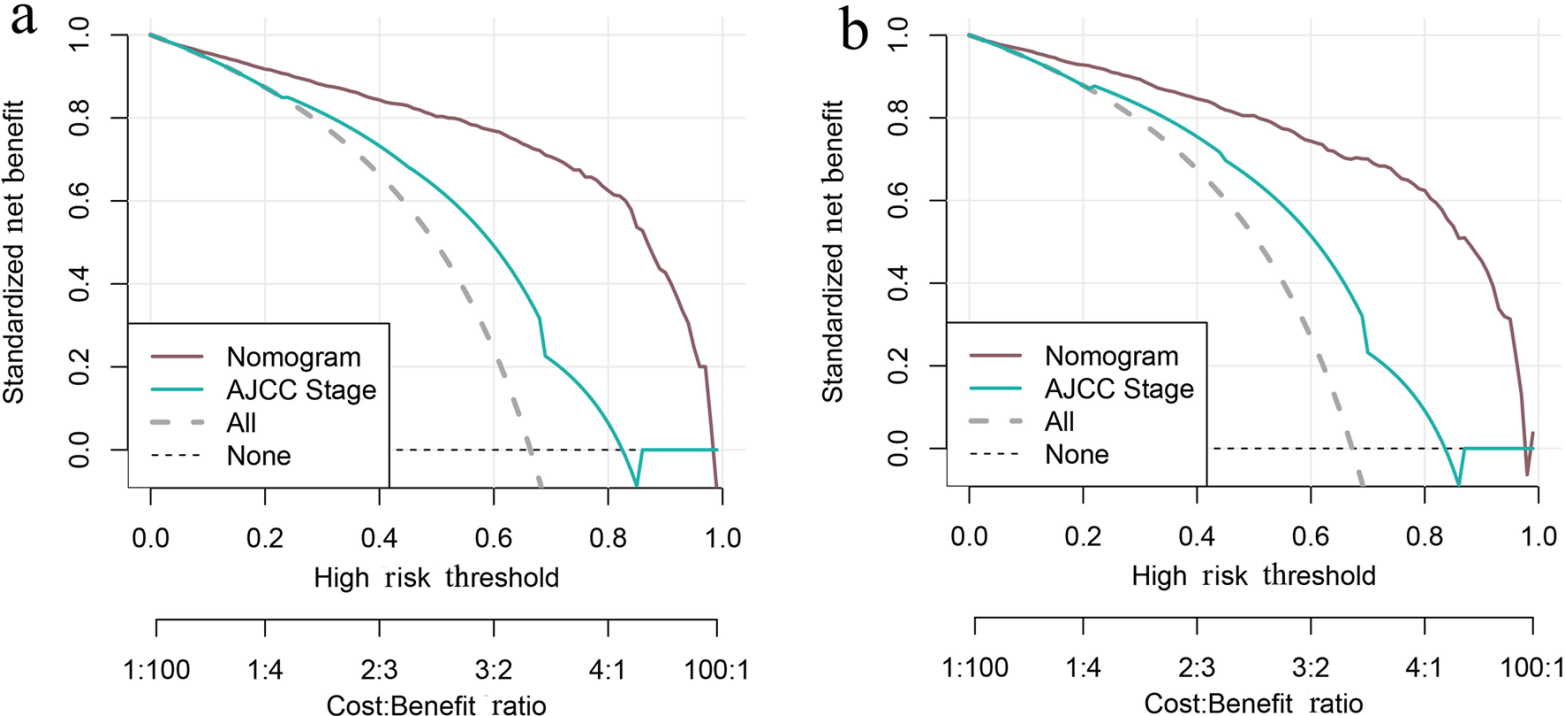
Decision curve analysis of the early death predictions from the nomogram and the AJCC stage in the validation set. (**a**) Training set. (**b**) Validation set

Figure 4a shows the ROC curves of the training set. The AUCs of the nomogram for 1-, 3-, and 6-month ED were 0.801, 0.812, and 0.880, respectively. All three values are above 0.5 and close to 1, suggesting that the nomogram gives reliable predictions of cancer-specific ED. When predicting survival time using the AJCC stage, the AUCs for 1, 3, and 6 months were 0.591, 0.631, and 0.715, respectively. Similarly, in the prediction of the prognosis using residual lesion size, the AUCs of 0.627, 0.655, and 0.770, respectively. For all three time intervals, AUCs were largest when using the nomogram, indicating that the nomogram had the most accurate prediction. We also compared the time dependence of the nomogram and AJCC stage prediction (Fig. 4b) and found that the nomogram performed better. Internal validation of the nomogram using a scatter plot of the actual probability (Y-axis) against the predicted probability (X-axis) showed that the calibration curves for all three time intervals are close to the 45° line, which indicates good calibration (Fig. 5a). In addition, Kaplan–Meier curves (Fig. 6a) were drawn to determine the difference in survival between high- and low-risk patients. Log-rank test was performed to differentiate the survival rate, and statistical significance was set at *P* < 0.0001. We also conducted a decision curve analysis (DCA) (Fig. 7a).

### Nomogram validation

The nomogram was validated in the validation set. Figure 4c shows the ROC curves of the nomogram, AJCC stage, and residual lesion size predictions of cancer-specific ED. When using nomogram, the AUCs for 1, 3, and 6 months were 0.798, 0.781, and 0.897; when using the AJCC stage, 0.602, 0.628, and 0.741; and when using residual lesion size, 0.635, 0.639, and 0.786, respectively. In the validation set, the AUC of the nomogram is still higher than those of the AJCC stage or residual lesion size predictions, indicating the superiority of the nomogram. This is also confirmed in the curves showing the time dependence of AUC (Fig. 4d) and the DCA curve (Fig. 7b). As shown in Fig. 5b, the calibration curves for all three time intervals are also close to the 45° line. Based on the median risk score derived from the nomogram, the Kaplan–Meier curve also showed significant differences between the low-risk and high-risk groups (Fig. 6b; validation set, *P* < 0.001). This indicates that the nomogram can effectively stratify risk. Internal validation of the nomogram in the validation set indicated good consistency between the predicted and observed values.

## Discussion

In this study, the ED rates of EOC at postoperative 1, 3, and 6 months were 9.98%, 18.73%, and 33.34%, respectively. Identifying patients at risk of ED is essential to reduce the burden on patients. Our results show that ED from ovarian cancer is mainly related the clinical factors AJCC stage, residual lesion size, chemotherapy, serum CA125 level, tumor size, number of lymph nodes examined, surgery of primary site, and age. Therefore, we constructed a nomogram that integrates these factors for predicting ED in patients with ovarian cancer.

Comparison of the ROCs and time-dependent AUCs showed that the nomogram had higher predictive power than the AJCC staging system. AJCC staging is commonly used to clinically evaluate ovarian cancer prognosis but is limited in that it cannot provide individualized predictions. Additional clinical factors, such as those included in our nomogram, are often ignored. Internal validation showed that the ED rate predicted with the nomogram is consistent with the actual ED rate. The C-index of the nomogram was 0.787, which represents a better degree of differentiation and ability to provide individualized prediction compared with the AJCC stage. In addition, both training and validation sets showed good consistency with observed values.

Nomograms have been widely used to evaluate the prognosis and death risk in patients with malignant tumor. Because of the lack of knowledge on specific symptoms, most patients of ovarian cancer are in advanced stage by the time of treatment. The main reasons for the high mortality in patients with ovarian cancer are recurrence and metastasis. Finally, most patients die of intestinal obstruction. Most studies on ED have focused on stage IV of ovarian cancer; therefore, a prognostic prediction model for early-onset ovarian cancer is needed. Our prediction model comprehensively considers all stages of ovarian cancer and suggests three “early” death periods (1, 3, and 6 months) as references. The prognosis of malignant tumors of the digestive tract is mostly related to race, marital status, and tumor location, which has been shown using nomograms [11–14]. Unlike most human cancers that spread through blood-borne metastasis, traditionally, EOC tumor cells were considered to metastasize directly by migrating through the peritoneal fluid to the omentum of the peritoneal cavity, highlighting its unique metastasis compared with that in lung cancer [15], breast cancer [16] and other cancers. In comparison, there are few cases of bone–brain–liver–lung metastases, but the variables in our nomogram include the “LN,” which stands for metastasis. However, recent studies have shown that ovarian cancer can also spread through blood-borne metastasis, and this may also help in identifying new opportunities for targeted drugs to treat EOC. Unlike most cancers that are associated with decreased differentiation, ovarian cancer becomes more highly differentiated during progression; therefore, our nomogram does not include “grade” [17]. The scope of surgery determines the prognosis to a large extent. Compared with the nomograms constructed for the ED of endometrial carcinoma [18] and uterine sarcoma [19], the nomogram we constructed for ovarian cancer comprehensively divides surgical methods rather than just estimating the prognosis based on surgery or not. In addition, we discussed the weightage of each variable and discrepancy in the weightage among all variables.

Chen et al. [20] studied all-cause and specific mortalities in ovarian clear cell carcinoma and constructed a nomogram with the prognostic factors age, laterality, organ metastasis, AJCC stage, number of lymphadenectomies, and chemotherapy. Yuan et al. [21] assessed lung metastasis incidence in ovarian cancer and proposed that stage, liver, bone, and brain metastases and TN stage are predictors of lung metastasis. The nomogram constructed by Mosgaard et al. [6] showed that the survival rate was higher for patients with chemotherapy, smaller residual cancer and tumor size, younger age, lower serum CA125 level, higher differentiation, debulking surgery, and more lymph node resections. Compared with existing nomograms to predict death risk in ovarian cancer, our nomogram included tumor size, residual size of cancer foci, and surgery of primary site, which are consistent with the factors affecting the prognosis of death risk in ovarian cancer, as discussed by Mosgaard et al. [6]. A recent article on ED in ovarian cancer studied advanced EOC (FIGO stages III and IV) [22]. However, our research included data on various stages of ovarian cancer, especially postoperatively. After performing univariate and multivariate regression analyses, pathological classification was not included in the construction of the nomogram. However, this recent article, which mainly studied advanced stage, focused on the pathological classification and metastases in the liver and lungs. In addition, our research is advanced in that it compared the advantages of using a nomogram to assess ED and discussed each variable in the model individually based on assessment of cancer stage and residual size of cancer foci. Since there is no clear time limit for ED, we predicted the probability of ED at postoperative 1, 3, and 6 months, instead of just for a specific ED time. The predictive model we constructed is more universal. In the present study, analysis and internal validation using ROC and DCA curves showed that the nomogram has good discrimination and calibration. Thus, the nomogram may be an effective tool to predict ED in patients with ovarian cancer, guide their individualized treatment, and improve their hospice care and quality of life.

Cancer stage is closely related to cancer survival and ED rates. In our nomogram, stage occupied the entire 100-point scale, with stage I being scored 0 and stage IV being scored 100. This implies that stage is an important independent influencing factor. This is consistent with the results of previous studies on prognostic evaluation models for pancreatic cancer and uterine sarcoma [19, 23]. The prognosis of stage III and IV patients is generally poor and mainly depends on the size of intraperitoneal metastases [24]. As stage progresses, the risk of death increases [25, 26]. A large retrospective cohort study based on the SEER database suggested that mortality in patients with stage III–IV ovarian cancer is as high as 10% in 1 year [27].

After stage, the residual size of cancer foci was the second most influential factor in this study. The larger the residual cancer foci, the higher the chances of ED, which is consistent with the findings of previous studies. Surgery is essential for ovarian cancer treatment, and postoperative residual tumor is one of the most relevant clinical prognostic factors [28–30]. Surgery aimed at minimizing tumor cells can lead to better outcomes. Complete tumor resection is considered a major predictor of survival [31]. Approximately, 20% of patients with advanced ovarian cancer survived for more than 12 years after treatment and were eventually effectively cured. Debulking surgery is performed to eliminate cancer cells and cancer foci as much as possible, preferably without significant residue. An article previously suggested that recovery depends on whether the combination of surgery and chemotherapy can effectively eliminate all cancer cells [32].

As an important adjuvant treatment for ovarian cancer, chemotherapy is commonly used to kill residual cancer foci and control or treat recurrent foci. In our study, chemotherapy was significantly correlated with prognosis, which is of great value in improving survival outcomes. Chemotherapy can reduce tumors and create conditions for surgery. Major tumor debulking surgery and platinum chemotherapy remain as the standard treatments for patients with stage III–IV EOC. Some patients with International Federation of Obstetrics and Gynecology stage III–IV ovarian cancer may benefit from neoadjuvant chemotherapy [33]. EOC patients (especially high-grade serous cancer) respond well to initial chemotherapy [3], with approximately 80% responding to neoadjuvant chemotherapy as an alternative treatment. Poly(ADP-ribose) polymerase inhibitors are one of the most studied, most effective, and least toxic drugs, and have, therefore, become one of the best targeted therapeutic options for treating recurrent EOC, especially in cases of platinum-sensitive recurrent ovarian cancer [34, 35]

Age and stage are independent risk factors for ovarian cancer prognosis [6]. In our study, age, lymph node examined, and tumor size were stratified, with cutoff values of 62 and 72 years for age, 2 for number of lymph nodes examined, and 90 mm for tumor size. In general, older patients are at higher risk of ovarian cancer and are more likely to have poor survival outcomes due to lower immune responses [36]. However, we observed that age was not highly significant, which may be related to the relatively conservative surgeries and pre- and postoperative chemotherapies given to young patients. In clinical practice, we often explore the pelvic and abdominal cavities of ovarian cancer patients to histologically examine suspected lesions and sites prone to metastasis and to clear the pelvic and abdominal para-aortic lymph nodes. Operation scope is determined according to the results of intraoperative exploration and frozen pathology examinations. The thoroughness of the first operation of EOC is closely related to the prognosis. Lymph node metastasis has an important effect on EOC prognosis. Some scholars suggest that lymph node dissection in advanced EOC may be used as a treatment. The number of lymph nodes examined and the resection of para-aortic lymph nodes may also be helpful [37]. In stage III, the subcategories IIIA and IIIB are based on the presence or absence of gross external pelvic peritoneal metastasis. However, this method is unable to distinguish the prognosis of patients with different numbers of lymphadenectomy in the same pathological stage. Therefore, we used X-tile to analyze the optimal cutoff value for the number of lymph nodes, which was subsequently included in the prediction after univariate and multivariate analyses.

Serum CA125 level is a high-sensitivity index for disease monitoring. Among patients with EOC, serum CA125 level is higher than the normal value, with more than 90% being consistent with the remission or deterioration of the disease. Increasing serum CA125 level is considered an important predictor of death [38] and is, therefore, important for predicting prognosis.

The primary ovarian focus in stage I patients are larger than those in stage III patients [24]. In addition, the ovarian foci in early ovarian cancer are more than twice as large as those in advanced ovarian cancer [39]. These support the fact that early and advanced ovarian cancer are two separate disease processes. Early tumors grow locally and do not spread, while advanced tumors that are relatively small are prone to spreading. It has been suggested that there may be a key substance differentiating the two processes; that is, the tumor in patients with advanced disease produces a substance that allows early-stage transmission. Without this substance, the tumor only grows locally.

The standard process for determining treatment in early EOC is clinical/surgical staging, which includes hysterectomy, bilateral ovariectomy, omentum resection, abdominal irrigation, and pelvic and para-aortic lymph node biopsy. Preserving the reproductive function means preserving the uterus and at least one side of the ovary. A study based on the SEER database found that fertility-sparing surgery was associated with an increased risk of death in women with advanced serous EOC [40]. However, some studies have found that the effect of fertility-sparing surgery on survival in stage I ovarian cancer is no worse than that of radical surgery. This suggests that specific histological subtypes have a greater effect on tumor prognosis than the retention of reproductive function. Radical surgery is unlikely to reduce the risk of recurrence of certain histological subtypes [41]. In a previous study, there was no significant difference in overall survival between stage I and radical surgery in EOC [42]. Prognosis may be more related to the natural history of the disease and the cancer type rather than to the specific type of surgery [43]. The nomogram we constructed refined the prognostic prediction by classifying the surgical modalities. Therefore, it is clear that the score for the difference of surgical type is non-significant.

This study has several limitations. First, our model did not include molecular markers that elucidate ovarian cancer mechanisms as these were not part of the SEER database. Many of these markers have been used to build predictive models for ovarian cancer [44], including 5 genes related to glucose metabolism [45] and 11 genes related to lipid metabolism [46]. MRPS12 may be a promising candidate for prognosis [47]. Second, several factors were also unavailable from the SEER database, including patients’ family history, underlying preoperative diseases, BMI, types of anesthetics, induction time, blood pressure, blood oxygen, heart rate fluctuations, cancer cell detection in pre- and postoperative ascites, thrombosis and surgical incision infection, specific preoperative and postoperative chemotherapy, chemotherapy times, and chemotherapeutic agents. Besides, not only was there no information provided regarding the time chosen to perform surgery (primary versus interval debulking) but the information whether operation was performed by an experienced surgeon or in a tertiary center was also missing. Third, we did not analyze humanistic and sociological factors such as income level and insurance, which have an important influence on the psychological and physiological aspects of ovarian cancer [8]. We also excluded economic status, education level, and follow-up by gynecologic oncologists, which are considered closely related to ovarian cancer prognosis. Lastly, our study is retrospective and has potential for selection bias since the data were extracted from the SEER database. Without external data validation, a more comprehensive prediction is impossible. Further studies combining our research data with those of others are needed for better prediction.

In conclusion, using a large cohort study, we identified several factors associated with ED in ovarian cancer and constructed a nomogram with better prognostic performance than the AJCC staging system. Our nomogram can be used in future clinical work as a more effective tool for screening high-risk patients. It may also play an important role in predicting ED from ovarian cancer and providing relatively reliable and individualized postoperative treatment advice to improve the quality of life of ovarian cancer patients.
